# Fatty Acids as a Tool to Boost Cancer Immunotherapy Efficacy

**DOI:** 10.3389/fnut.2022.868436

**Published:** 2022-06-23

**Authors:** Annemarie J. F. Westheim, Lara M. Stoffels, Ludwig J. Dubois, Jeroen van Bergenhenegouwen, Ardy van Helvoort, Ramon C. J. Langen, Ronit Shiri-Sverdlov, Jan Theys

**Affiliations:** ^1^Department of Precision Medicine, GROW-Research School for Oncology and Reproduction, Maastricht University Medical Center+, Maastricht, Netherlands; ^2^Department of Genetics and Cell Biology, NUTRIM-School of Nutrition and Translational Research in Metabolism, Maastricht University, Maastricht, Netherlands; ^3^Danone Nutricia Research, Utrecht, Netherlands; ^4^Department of Pharmacology, Faculty of Science, Utrecht Institute for Pharmaceutical Sciences, Utrecht University, Utrecht, Netherlands; ^5^Department of Respiratory Medicine, NUTRIM-School of Nutrition and Translational Research in Metabolism, Maastricht University Medical Center, Maastricht, Netherlands

**Keywords:** cancer, immunotherapy, fatty acid, SCFAs, PUFAs

## Abstract

Although immunotherapy represents one of the most potent therapeutic anti-cancer approaches, only a limited number of patients shows clinical benefit. Recent evidence suggests that patients' nutritional status plays a major role in immunotherapy outcome. Fatty acids are essential in a balanced diet and well-known to influence the immune response. Moreover, short-chain fatty acids (SCFAs) show beneficial effects in metabolic disorders as well as in cancer and polyunsaturated fatty acids (PUFAs) contribute to body weight and fat free mass preservation in cancer patients. In line with these data, several studies imply a role for SCFAs and PUFAs in boosting the outcome of immunotherapy. In this review, we specifically focus on mechanistic data showing that SCFAs modulate the immunogenicity of tumor cells and we discuss the direct effects of SCFAs and PUFAs on the immune system in the context of cancer. We provide preclinical and clinical evidence indicating that SCFAs and PUFAs may have the potential to boost immunotherapy efficacy. Finally, we describe the challenges and address opportunities for successful application of nutritional interventions focusing on SCFAs and PUFAs to increase the therapeutic potential of immunotherapeutic approaches for cancer.

## Introduction

According to the world health organization (WHO), cancer is the second leading cause of death globally. Worldwide, cancer accounted for nearly 10 million death in 2020 and the cancer burden further continues to grow (1, 2). Immunotherapy, a treatment that utilizes the immune system in order to help the body to fight cancer, represents one of the most promising novel treatment approaches. A variety of different immunotherapeutic strategies are currently being used, including immune checkpoint inhibitors (3), immuno-cytokines (4), monoclonal antibodies (5), adoptive T or NK cell based therapies (6, 7) and cancer vaccines (8). To improve therapeutic outcome, immunotherapy is often combined with other treatments such as chemotherapy or radiation (9).

However, despite long-lasting effects of immunotherapy in some responders (4, 10), disease control occurs in only a small subset of patients (11–13). For example, <13% of the eligible patients for immune checkpoint inhibitor therapy in the U.S. actually benefit from this treatment (11). This low response rate can in part be explained by the fact that a small, but significant proportion of patients receiving immunotherapy develop immune-related adverse effects that dictate cessation of treatment (14). However, in the majority of the patients, the underlying reasons for the lack of response to immunotherapy are unknown. Various mechanisms have been proposed, such as low programmed death-ligand 1 (PD-L1) expression on tumor cells limiting efficacy of immune checkpoint inhibitors (15) and low mutational burden in combination with downregulation of human leukocyte antigen (HLA) proteins. The latter disrupts the process of antigen presentation of tumor cells, thereby hindering effective T cell recognition, eventually leading to failing of T cell-based immunotherapeutic approaches (16). In addition, the tumor microenvironment (TME) influences immunotherapy responses, e.g., a hypoxic TME impairs anti-tumor immunity and has been suggested to suppress the efficacy of immunotherapy (17). Also, infiltration in the TME of regulatory T cells, myeloid-derived suppressor cells (MDSCs) and M2 tumor-associated macrophages (TAMs) is associated with immunosuppression (16). Moreover, the efficacy of immunotherapy is dependent on a competent immune system, but the latter can be compromised due to multiple host factors, including malnutrition, a problem often encountered in cancer patients (18, 19).

Several epidemiological studies have reported an association between nutritional and metabolic status of cancer patients and responsiveness to immunotherapy. For instance, a low prognostic nutritional index (PNI) has been reported as an independent predictor of short time to treatment failure in lung cancer patients treated with the anti-PD-L1 immune checkpoint inhibitor Atezolizumab (20). In another cohort of lung cancer patients, malnutrition parameters, such as hypoalbuminemia and significant weight loss, have been associated with decreased immunotherapy efficacy (21). Moreover, clinical data from lung cancer and melanoma patients have indicated that cachectic cancer patients appear refractory to immune checkpoint inhibitor therapy (22). In contrast, obesity has been associated with improved responses to immune checkpoint blocking agents in cancer patients (23). Obesity results in increased inflammation and immunosenesence, tumor progression and PD-1-mediated T cell dysfunction which is driven, at least in part, by elevated leptin levels (24). Elevated levels of PD-1 are correlated with increased T cell exhaustion, but also facilitates the success of anti-PD-1 checkpoint therapy, contributing to increased overall survival of obese cancer patients treated with anti-PD-1 antibodies (24). Thus, evidence of an association between nutritional status and immunotherapy efficacy is arising and the underlying mechanisms explaining to what extent the nutritional status is involved in the responsiveness to immunotherapy are becoming increasingly clear.

Fatty acids (Supplementary Box 1) are essential in a balanced diet and dietary fatty acids are well-known to influence the nutritional status as well as the immune response of cancer patients (25, 26). Specifically, oral nutritional supplementation containing omega-3 polyunsaturated fatty acids (*n*-3 PUFAs) resulted in preservation of body weight and fat free mass in lung cancer patients (25). Moreover, nutritional intervention with a specific diet rich in *n*-3 PUFAs, reduced serum levels of inflammatory mediators in cancer patients receiving radiotherapy (26). The role of omega-6 PUFAs (*n*-6 PUFAs) on inflammation is more controversial. Although in general high intake of *n*-6 PUFAs has been linked to increased inflammation, some studies also suggest that specific *n*-6 PUFAs can actually decrease inflammation (27). Finally, as a separate class of fatty acids, short-chain fatty acids (SCFAs), formed in the gut upon fermentation of dietary fibers, are known for their anti-inflammatory properties (28) and show beneficial effects in metabolic disorders as well as in cancer (29–31).

Overall, epidemiological evidence associating nutritional status to immunotherapy outcome is increasing and the beneficial effects of specific types of SCFAs and PUFAs on nutritional status, metabolism and the immune response are well-established (25–31). In the next paragraphs, the evidence supporting the potential use of dietary interventions with SCFAs and PUFAs to enhance immunotherapy efficacy will be discussed.

## SCFAs and PUFAs Potentially Enhance Immunotherapy Efficacy

### Epidemiological Data Indicate That SCFAs Associate With Response to Immunotherapy

Epidemiological studies specifically investigated the relationship between serum and fecal SCFA concentration and immunotherapy response. In that context, Nomura et al. demonstrated that high concentrations of fecal acetic, propionic, butyric and valeric acids were associated with longer progression-free survival in patients with solid tumors receiving the anti-PD-1 antibodies Nivolumab or Pembrolizumab (32). In line with the data presented by Nomura et al., metabolomics profiling of the gut microbiota from patients with non-small cell lung cancer (NSCLC) receiving Nivolumab showed that propionate and butyrate were significantly associated with long-term beneficial effects (33). However, Coutzac et al. reported an inverse relation between serum SCFA levels and outcome in melanoma patients receiving the anti-CTLA-4 antibodies Ipilimumab; patients with lower serum levels of butyrate and propionate demonstrated longer progression free survival (34). These findings may be the result of the complex interplay between production and absorption of SCFAs in the gastrointestinal tract. Taken together, the results of these studies suggest that fecal and/or serum SCFAs concentrations associate with response to immunotherapy.

### *In vitro* Data Indicate That SCFAs and *n*-3 PUFAs Enhance Immunotherapy Efficacy

A plethora of *in vitro* studies has indicated the potential of SCFAs to improve immunotherapy efficacy via enhancing the immunogenicity of cancer cells. Already in 1994, it was shown that colon adenocarcinoma cells enhanced the expression of major histocompatibility complex 1 (MHC-1) and intercellular adhesion molecule 1 (ICAM-1) upon butyrate exposure, which makes tumor cells more sensitive to cytotoxic lymphocytes-mediated killing (35). More recently, acetate has been shown to reduce the expression of CD155 on colorectal cancer cells (36). CD155 is a ligand for the inhibitory receptor T cell immunoreceptor with Ig and ITIM domains (TIGIT) expressed on natural killer (NK) cells, T cells and dendritic cells (DCs) and is frequently upregulated in malignant cells (37–39). Downregulation of CD155 on cancer cells has been suggested to enhance CD8+ T cell effector responses toward cancer cells (36). Andresen et al. and Hogh et al. (40, 41) both demonstrated that propionate induced the expression of the natural killer group 2D (NKG2D) ligands MHC class I polypeptide-related sequence A/B (MICA/B) on cancer cells. Activated NK and T cells recognize these MICA/B positive cells via the NKG2D receptor, followed by elimination of the target cell upon ligand-receptor coupling (42). Altogether, these *in vitro* data imply that SCFAs are capable of sensitizing cancer cells to immunogenic responses, potentiating the effects of immunotherapeutic approaches used to combat cancer.

Immunotherapy removes the break on the immune system, potentially causing a range of undesired inflammatory side-effects (43). Inflammation at barrier organs, including the gastrointestinal mucosa, is a common sign of toxicity in patients treated with immune checkpoint blockers (44). The gastrointestinal mucosa has an important role in controlling pathogenic organisms, while maintaining adequate permeability for nutrient absorption (45). A disruptive intestinal barrier can cause microorganisms to translocate into the bloodstream leading to adverse effects (46). In the context of fatty acids, *in vitro* studies have indicated that SCFAs significantly improve intestinal barrier function, measured by transepithelial electrical resistance (TEER) (47). In agreement, Nielson et al. found that butyrate at physiologically relevant concentrations (1-10 mM) significantly improved epithelial barrier function in E12 human colon cells (48) and Peng et al. also confirmed that butyrate (2 mM) improves intestinal barrier function (49). Together, these data indicate that SCFAs can improve gut barrier function and thereby might suppress the immune-mediated toxicities often induced by immunotherapy.

Another possible side effect of immunotherapy is cytokine storm syndrome, which can be harmful as it can interfere with body functions and in severe cases even can lead to organ failure and death (50). Park et al. demonstrated that acetate promotes T cell differentiation into both effector T cells producing IL-17 and interferon γ (IFNγ) or regulatory T cells producing IL-10, depending on the cytokine milieu (51). It has been proposed that butyrate and propionate, but not acetate, modulate cytotoxic T cell activation by inhibiting DC secretion of IL-12. Importantly, butyrate and propionate supported a more tolerogenic immune activation of the innate immune system instead of a pro-inflammatory response in the gut (52). The results of these studies highlight the potential of SCFAs to provide a balance between inflammation and immunity, and it is tempting to speculate that these SCFAs may prevent the cytokine storm syndrome often induced by T cell based immunotherapy.

Similar as for SCFAs, *in vitro* studies have suggested that *n*-3 PUFAs might contribute to suppression of exacerbated inflammatory cytokine production by immune cells. Long chain PUFAs, present in membrane phospholipid, are released by phospholipases and serve as substrate for cyclooxygenase isozymes and 5-lipoxygenase and are precursors for different prostaglandins, leukotrienes, thromboxanes and other eicosanoids. The relative abundance of *n*-3 and *n*-6 PUFAs and the respective lipid species within these categories determine the eicosanoid lipid mediators species and the respective effect on immune function and their pro- or anti-inflammatory potential. Where in general the eicosanoids derived from the *n*-6 fatty acid (arachidonic acid, ARA) have a high pro-inflammatory potential, the species derives from *n*-3 fatty acid (eicosapentaenoic acid, EPA and docosahexaenoic acid, DHA) have low or even anti-inflammatory properties. For instance, Hao et al. showed that EPA treatment reduced lipopolysaccharide (LPS) or prostaglandin E 2 (PGE-2)-induced expression of IL-6 and tumor necrosis factor α (TNFα) and increased the expression of IL-10 in both macrophages and hepatocytes (53). While EPA reduces these inflammatory responses, its direct anti-carcinogenic effects on tumor cells is preserved (53, 54). Hence, *n*-3 PUFA supplementation has been suggested as a useful addition for adoptive T cell therapy (54). These studies propose that dietary interventions focusing on *n*-3 PUFAs could also be beneficial to prevent or diminish the cytokine storm.

The efficacy of immunotherapy has been shown to be dependent on the TME. For instance, a hypoxic TME impairs anti-tumor immunity, induces T cell exhaustion and has been suggested to suppress the efficacy of immunotherapy (17). Moreover, infiltration of immunosuppressive cells, such as regulatory T cells, MDSCs and M2 TAMs into the TME is associated with immunosuppression, potentially affecting immunotherapy efficacy (16) and a recent review highlighted a major role for cancer-associated fibroblasts in the TME in promoting immunotherapy resistance (55). Multiple studies indicate that SCFAs, *n*-3 and *n*-6 PUFAs alter the TME. Specifically, butyrate inhibited the hypoxia-induced induction and activity of hypoxia-induced factor 1α (HIF-1α) in HT1080 human fibrosarcoma cells and butyrate also suppressed HIF-1α and vascular endothelial growth factor (VEGF) expression in vascular endothelial cells in hypoxic conditions *in vitro* (56). Similarly, DHA supplementation *in vitro* resulted in decreased HIF-1α total protein levels and transcriptional activity in the malignant breast cell lines, but not in the non-transformed cell line (57). Thus, SCFAs and *n*-3 PUFAs may exert relevant anti-cancer effect in a hypoxic TME. In addition, as described before, depending on the cytokine milieu, acetate promotes a pro-inflammatory TME *via* enhancing effector T cell function or suppresses inflammation *via* promoting differentiation of regulatory T cells (51). Also, in general, increased dietary *n*-6 PUFA consumption is associated with a pro-inflammatory TME, while *n*-3 PUFA rich diets suppress inflammation (the effects of dietary PUFAs on immune cells in the TME has been extensively reviewed by Khadge et al.) (58). Furthermore, SCFAs as well as *n*-3 PUFAs have been shown to inhibit fibroblast matrix metalloproteinase secretion into the TME (59, 60). However, it remains to be studied whether SCFAs, *n*-3 and *n*-6 PUFAs influence the outcome of immunotherapy via modulation of the TME.

In addition, specific PUFAs have been described to enhance immunotherapy outcome via other mechanisms. For example, it has been shown that DHA can enhance the anti-proliferative as well as the apoptotic effect of tumor necrosis factor-related apoptosis inducing ligand (TRAIL—an immune-cytokine used as immunotherapy) specifically for cancer cells (61). Kumar et al. demonstrated the potential of ARA to enhance the capacity of DCs to exhibit increased *in vitro* and *in vivo* chemotaxis accompanied with better stimulatory and cytotoxic T cell activity as well as a favorable T helper cell 1 (Th1) cytokine profile. These results highlight the potential of ARA to enhance DC capacity for DC-based vaccines for cancer immunotherapy (62).

### Preclinical *in vivo* Data on the Effects of SCFAs and PUFAs on Immunotherapy Outcome Are Inconclusive

Several *in vivo* studies investigated the effects of SCFAs on the efficacy of immunotherapy. One of the earliest observations, in 1994, indicated that intraperitoneally injected butyrate significantly enhanced the immune-mediated effects of recombinant IL-2 treatment in a subcutaneous adenocarcinoma rat model (35). In a more recent study, mice bearing melanoma or pancreatic tumors were treated with an adoptive T cell therapy approach. The results showed that the *in vivo* anti-tumor immunity of transferred cytotoxic T cells was ameliorated when cultured *ex vivo* in presence of butyrate and pentanoate. The improved *in vivo* cytotoxic T cell response was explained by histone deacetylase activity (HDAC) inhibiting capacity of butyrate and pentanoate, which enhanced the expression of effector molecules (TNFα and IFNγ) produced by cytotoxic T cells (63). However, it should be noted that the effect of cytotoxic T cells transferred in tumor bearing mice was absent when pentanoate was administrated *in vivo* via injections (63). The exact mechanism has not yet been elucidated, but one possible explanation might be that *in vivo* pentanoate administration, similarly as butyrate, propionate and acetate (64–66), not only improves the function of effector T cells, but also promotes T cell differentiation into regulatory T cells (51), thereby resulting in no overall beneficial effect of the adoptive T cell therapy approach *in vivo*. Yet, the promoting or suppressing function of SCFAs on anti-cancer T cell mediated cytotoxicity *in vivo* requires further examination, especially since another study did not observe any changes in the frequency of regulatory T cells in tumors upon oral butyrate administration in a subcutaneous colon cancer mouse model (67). Actually, in this study, oral butyrate administration even boosted the anti-tumor responses of CD8+ effector T cells *in vivo* (67). Altogether, from these data it is speculated that SCFAs can push the immune response in 2 direction, either toward enhanced CD8+ effector T cell functioning or toward increased differentiation of immuno-suppressive regulatory T cells. Which direction is activated by the SCFAs most likely depends on the cytokine environment (51). Although these studies did not show directly a beneficial effect of SCFAs on adoptive T cell therapy efficacy *in vivo*, these data imply a role for SCFAs in *ex vivo* culturing of T cells used in adoptive T cell therapies (63). Moreover, several preclinical mouse studies have investigated the combinatory effects of SCFAs and immune checkpoint inhibitors. Han et al. demonstrated that oral administration of inulin, a dietary fiber serving as a nutrient source for the gut bacteria which generate SCFAs, modulates the gut microbiome composition. Consequently, the anti-tumor activity of anti-PD-1 antibodies was amplified in murine models of colon cancer and melanoma (68). In agreement, mice bearing melanoma tumors treated with anti–PD-1 therapy in combination with a fiber-rich diet demonstrated delayed tumor outgrowth compared to mice receiving a fiber-poor diet. The therapeutic gain observed in the mice receiving the fiber-rich diet might partly be explained by the significantly higher levels of propionate observed in the stool samples (69). Furthermore, anti-PD-1 antibody efficacy was largely impaired in MC38-tumor bearing mice receiving fecal microbiota transplantation (FMT) from newly diagnosed colorectal cancer patients compared to mice receiving FMT from healthy controls. Remarkably, dietary pectin, a soluble fiber that is fermented in many metabolites in the gut, including SCFAs, could reverse the poor efficacy of anti-PD-1. Follow-up experiments indicated that supplementation of butyrate (but not acetate) in the drinking water, instead of pectin, was already sufficient to result in synergistic therapeutic effects when combined with immune checkpoint inhibitor therapy (70). Although these studies suggest a role for SCFAs in supporting immune checkpoint inhibitor therapy, it was previously shown that butyrate supplementation reduced the efficacy of anti-CTLA-4 antibodies in multiple tumor mouse models, by inhibiting the upregulation of the co-stimulatory molecules CD80/CD86 on dendritic cells (34). In line, no beneficial effect of anti-PD-1 treatment in combination with pentanoate injections was observed in a subcutaneous mouse model for melanoma (63). The authors did not explore the reason of these negative data, but given the small number of mice and the large variation in the data, the power of this experiment may have been too low to reach statistically significant differences. Overall, currently published *in vivo* studies investigating the effects of SCFAs on the outcome of immunotherapy provide contradictive information. The opposing results obtained in the different studies could be related to differences in the experimental design such as concentrations, route of administration of the SCFAs or different dietary fibers fermentable in SCFAs, different response read-outs as well as different types of immunotherapy treatment. Therefore, improved standardization of intervention designs, and use of appropriate experimental models will further facilitate systematic evaluation of the effects of SCFAs on the outcome of immunotherapy.

In contrast to SCFAs, preclinical *in vivo* studies on the direct effects of dietary PUFAs on the outcome of immunotherapeutic approaches in cancer are lacking. However, several preclinical cancer models show that these lipids can modulate immune responsiveness. For example, in a mouse model for obesity-associated breast cancer, a high fat diet (HFD) in combination with fish oil resulted in a reduction of inflammatory markers (TNFα, IL-6) and in an increase of the anti-inflammatory marker IL-10, compared to HFD alone (71). Additionally, experimental research in colon cancer tumor bearing cachectic mice has revealed that intervention with a diet rich in *n*-3 PUFAs reduced the inflammatory state and improved immune competence (72). Furthermore, in a mouse model of castrate-resistant prostate cancer, administration of a diet rich in *n*-3 PUFAs inhibited the function of M2 tumor associated macrophages (TAMs) (73). Opposite to *n*-3 PUFAs, diets rich in saturated fatty acids (SFAs) promote an immunosuppressive TME, conceivably via stimulating chronic low-grade inflammation. For example, Liu et al. demonstrated that SFAs enhance the differentiation of pro-tumorigenic TAMs. In this study, breast tumor-bearing mice were fed a high fat diet consisting of either cacao butter (rich in SFAs) or fish oil (rich in *n*-3 PUFAs). Fish oil resulted in uncoupled obesity-associated tumor growth and reduced the number of pro-tumoral TAMs, whereas cacao butter enhanced the differentiation of pro-tumoral TAMs (74). In addition, the *n*-6 PUFA ARA, which can be converted into several prostaglandins including PGE-2, stimulated the accumulation of myeloid-derived suppressor cells (MDSC) inhibiting immunosurveillance in the TME (75). Overall, these data suggest that *n*-3 PUFAs can reduce chronic low-grade inflammation in cancer, while SFAs and *n*-6 PUFAs lead to an immunosuppressive TME via stimulation of chronic low-grade inflammation. Nevertheless, it remains to be addressed whether these SFAs, *n*-3 and *n*-6 PUFAs influence the outcome of immunotherapy. Here as well, standardization of intervention designs, and selection of appropriate experimental models will expedite systematic exploration of the potential of *n*-3 or *n*-6 PUFAs to contribute to clinical efficacy of immunotherapy.

In addition to direct effects of PUFAs on immune cells, PUFAs can also influence the immune response by modulating the gut microbiome. Preclinical evidence has shown that *n*-3 PUFAs, especially EPA and DHA, can modify the gut microbiota composition in several rodent models in a beneficial manner by increasing the intestinal population of Bifidobacteria (76, 77), Akkermansia muciniphila bacteria (77, 78) and Firmicutes bacteria (79). Contrary, a diet high in *n*-6 PUFAs has been shown to induce gut microbiome dysbiosis resulting in a marked reduction of Firmicutes, Clostridia and Lachnospiraceae bacterial presence while stimulating growth of Bacteroidetes and Deferribacteraceae bacteria and the pro-inflammatory Mucispirillum schaedleri and Lactobacillus bacteria (80). In line, supplementation of high-fat diets rich in *n*-6 PUFA to aged mice caused dysbiosis resulting in intestinal inflammation by promoting bacterial overgrowth while depleting microbes from the Bacteroidetes and Firmicutes phyla (81). Although evidence is arising that the microbiota composition is essential for determining immunotherapy outcome, there is currently no consensus what type of microbiota composition or which microbial species are robustly associated with clinical responses; while one study reported an association between high abundance of Bifidobacterium longum, Collinsella aerofaciens, and Enterococcus faecium and improved responses to immunotherapy (82), other studies reported an association between higher abundance of microbes from the Verrucomicrobiota and Firmicutes phyla and enhanced immunotherapy responses (83, 84). Thus, despite recognition of prebiotic properties of PUFAs, the effects of PUFAs on immunotherapy outcome remain ambiguous.

### Fermentable Fibers and *n*-3 PUFAs Have the Potential to Enhance Clinical Immunotherapy Efficacy

Data on specific fatty acid tailored dietary intervention studies to explore the effect on immunotherapy responsiveness in cancer patients are not yet available. However, recently, a cohort study investigated whether intake of dietary fiber (fermenting into SCFAs) affects clinical outcome of melanoma patients treated with different immune checkpoint inhibitors. The patients reporting sufficient dietary fiber intake, using the National Cancer Institute Dietary Screener Questionnaire, demonstrated a significantly longer progression-free survival compared to patients reporting insufficient dietary fiber intake (69). To evaluate whether dietary fiber intake and probiotic use may synergistically affect clinical outcomes in these melanoma patients treated with immune checkpoint inhibitors, the study compared levels of fiber intake and probiotic use in this patient population. Strikingly, longest progression-free survival was observed in patients reporting sufficient dietary fiber intake without probiotic use (69). These findings suggest that use of commercially available probiotics consumed by this study population is not beneficial in the setting of immune checkpoint inhibitors, while dietary fiber interventions synergistically enhance immunotherapy efficacy potentially by supporting a diverse microbiome and increasing SCFA content. Along this line, a phase 2 clinical trial (NCT04645680), aiming to investigate the effects of dietary fiber intervention on the structure and function of the gut microbiome in patients with melanoma treated with Pembrolizumab or Nivolumab, is currently recruiting patients.

Although direct clinical evidence regarding the effects of PUFAs on immunotherapy outcome is lacking, multiple clinical intervention studies in cancer patients indicate that *n*-3 PUFAs modulate immune responsiveness by reducing chronic low-grade inflammation (85). For example, the role of EPA and DHA on inflammatory and oxidative status in patients with NSCLC treated with chemotherapy was investigated in a multicenter randomized double-blinded control trial. Results indicated that dietary administration of these *n*-3 PUFAs decreased the levels of oxidative stress as well as the production of the pro-inflammatory mediators C-reactive protein (CRP) and IL-6 (86). Furthermore, increased concentrations of EPA and DHA, as a result of consumption of a medical food rich in fish oil, protein, and leucine, reduced serum levels of the inflammatory mediator PGE-2 in a randomized clinical trial for patients receiving radiotherapy (26). Overall, these clinical intervention studies indicate that *n*-3 PUFAs have anti-inflammatory effects in cancer patients. However, as indicated before, it remains unclear how these *n*-3 PUFAs influence the outcome of immunotherapy.

Several clinical intervention studies have associated *n*-3 PUFA rich diets with modulation of the gut microbiome in humans. For example, healthy volunteers receiving *n*-3 PUFA rich diets for 8 weeks, reversibly increased the abundance of the SFCA producing bacteria Bifidobacterium, Roseburia and Lactobacillus in the gut (87). In addition, type 2 diabetes patients treated with a diet enriched with 100 g sardines 5 days a week for 6 months demonstrated a decreased Firmicutes/Bacteroidetes ratio at the end of the study compared to standard diet. Both dietary interventions decreased phylum Firmicutes concentrations (88). These clinical studies, similarly as for the *in vivo* animal data, indicate that *n*-3 PUFAs may modulate the gut microbiome beneficially. However, since there currently is no consensus what type of microbiota composition or which microbial species are robustly associated with clinical responses to immunotherapy, the effects of *n*-3 PUFAs on immunotherapy outcome in cancer patients remains uncertain.

## Challenges, Opportunities and Future Directions

**Figure 1:**
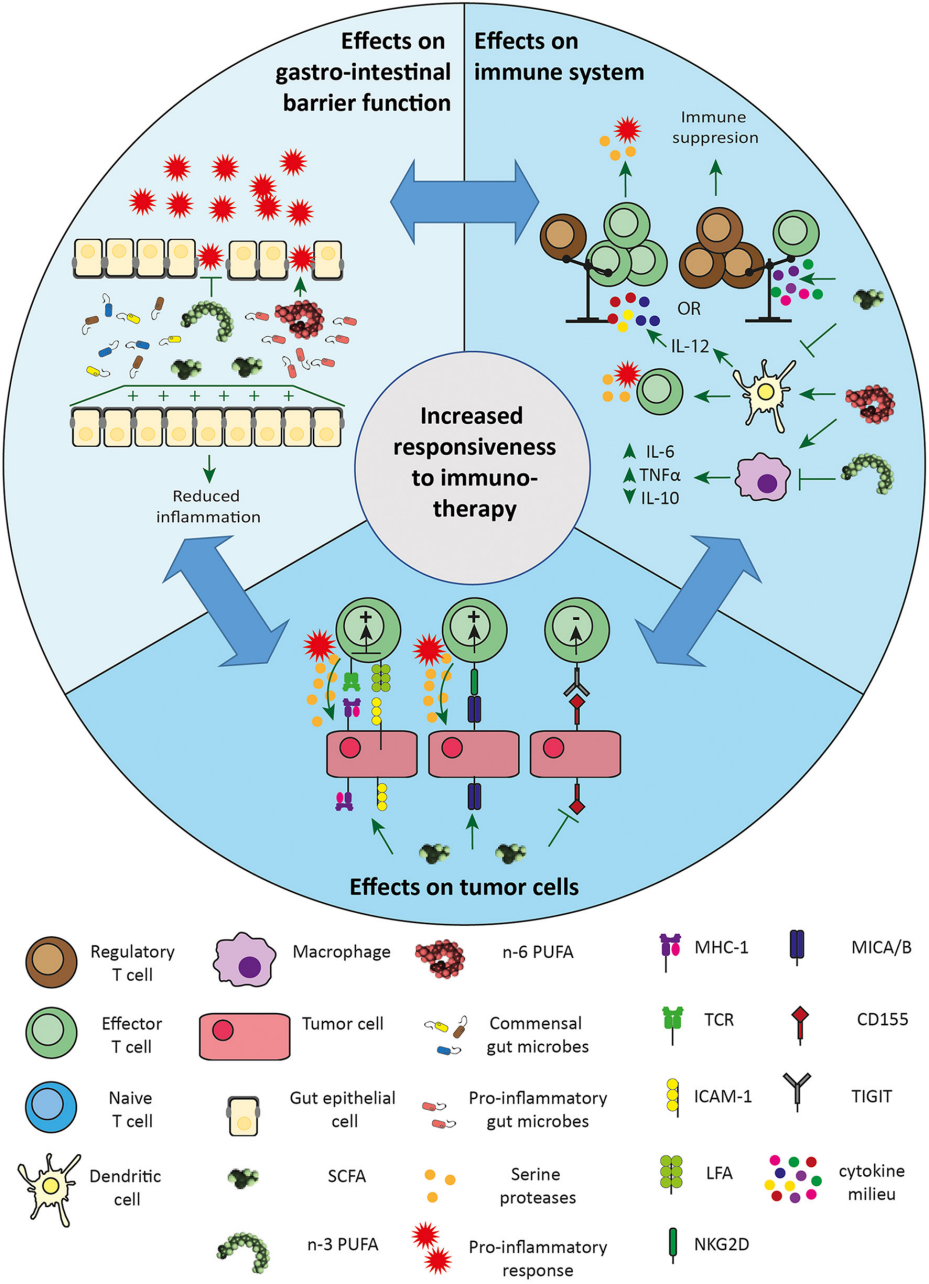
Proposed mechanisms through which SCFAs and PUFAs enhance immunotherapy efficacy. Effects on gastric barrier: SCFAs can improve gut barrier function and have, as well as *n*-3 PUFAs, anti-inflammatory effects. Diets rich in *n*-3 PUFAs have a beneficial effect on the gut microbiome. Both have beneficial effects on gastro-intestinal functioning, thereby reducing immune-mediated toxicity and may enhance to immunotherapy outcome, as the need of cessation of treatment is lower. Diets rich in *n*-6 PUFAs lead to dysbiosis accompanied with pro-inflammatory effects. Effects on immune system: SCFAs promote T cell differentiation in both effector T cells and regulatory T cells, depending on the cytokine milieu. SCFAs also inhibit IL-12 secretion from dendritic cells modulating effector T cell activation. Contrary, *n*-6 PUFAs enhance dendritic cell capacity to stimulate cytotoxic T cell activity, directly reducing tumor growth. *n*-6 PUFAs' pro-inflammatory effects occur mainly via stimulation of macrophages contributing to chronic low-grade inflammation. In contrast, *n*-3 PUFAs suppress inflammation via reducing IL-6 and TNFα and increasing IL-10 production. Effects on tumor cells: SCFAs enhance the expression of MHC-1 and ICAM-1 on tumor cells, making them more sensitive to cytotoxic lymphocytes-mediated killing. SCFAs also induce the expression of MICA/B on tumor cells, making them a target for effector T cells via the NKG2D receptor. SCFAs reduce the expression of CD155 on tumor cells, inhibiting the interaction with TIGIT expressed on effector CD8+ T cells. ICAM-1, intercellular adhesion molecule 1; IL-6, interleukin 6; IL-10, interleukin 10; IL-12, interleukin 12; LFA, lymphocyte function-associated antigen 1; MHC-1, major histocompatibility complex 1; MICA/B, MHC class I polypeptide-related sequence A/B; NKG2D, natural killer group 2D; *n*-3 PUFAs, omega 3 polyunsaturated fatty acids; *n*-6 PUFAs, omega 6 polyunsaturated fatty acids; SCFAs, short chain fatty acids; TCR, T cell receptor; TIGIT, T cell immunoreceptor with Ig and ITIM domains; TNFα, tumor necrosis factor α.

In this review we have described the influence of dietary intervention with SCFAs and dietary fibers that are fermented in SCFAs on immunotherapy efficacy. Proposed mechanisms through which SCFAs enhance immunotherapy efficacy include sensitization of cancer cells to immunogenic responses, improved gut barrier function and enhanced cytotoxic T cell functioning (see Figure 1). Moreover, recent clinical data indicate that fiber rich diets are beneficially impacting immunotherapy outcome, potentially via supporting fiber fermentation, which yields increased content of SCFAs or by increasing the gut microbiota diversity. Overall, dietary fiber or SCFA administration holds the potential to improve immunotherapy efficacy. Yet, most evidence is rather speculative and direct proof for an effect of SCFAs on immunotherapy outcome is relatively sparse and sometimes even contradictory. Therefore, to fully understand the mechanisms underlying the effects of different SCFAs on immunotherapy efficacy, more research will be essential.

We have further depicted the impact of dietary intervention with PUFAs on immunotherapy outcome. The currently available data indicate that for cancer patients with elevated systemic chronic low-grade inflammation, e.g., obese patients, a diet rich in *n*-3 PUFAs might be preferred above a *n*-6 PUFA rich diet which promotes an immunosuppressive TME by stimulating chronic low-grade inflammation. Similar to patients with obesity, malnourished patients suffering from sarcopenia or cachexia often have chronic inflammation leading to immune senescence and may also benefit from intervention with *n*-3 PUFAs to reduce chronic inflammation and thereby potentially improve immune competence and immunotherapy efficacy. Nonetheless, whether in these malnourished patients, consumption of anti-inflammatory *n*-3 PUFAs restores, stimulates or actually further inhibit immunotherapy efficacy is currently unknown. One could also argue that elevated *n*-6 PUFA levels, which are regarded as more pro-inflammatory, may support the immune activating properties of immunotherapy in patients with immune senescence. Thus, depending on the nutritional status of cancer patients, either *n*-3 or *n*-6 PUFAs may contribute to enhance immunotherapy efficacy, awaiting further validation in follow-up experiments.

The collective, sometimes contradictive or inconclusive evidence available on the use and influence of dietary intervention with SCFAs or PUFAs to improve therapy outcome, highlights the importance of metabolic profiling and personalized medicine in this context. It will be essential to develop tailored diets: a single recommended diet for all cancer patients treated with immunotherapy most likely not exist due to the variability in metabolism of lipids and immune responses. In that context, nutritional status or patients' body composition should be taken into account. Obese individuals for instance, have significantly higher fecal SCFA concentrations with a similar fiber intake, compared to lean individuals (89). Also, malnourished patients may require a different route of administration of the dietary intervention then obese patients. There are different ways to administer diets according to the patients' needs, including classical oral intake via a dietary regimen, but also supplementation with enriched oral nutritional supplements, capsules or concentrated parenteral emulsions or injections, specifically for patients who cannot adhere to the recommended intake via the classical way. Since personalized nutritional interventions are relatively feasible, this approach holds the potential to extend the clinical benefit of immunotherapeutic approaches to many different populations who currently do not benefit from this treatment. Yet, several challenges need to be overcome before fatty acid focused dietary regimens can be integrated in standard of care. First of all, dietary interventions require sufficient consumption and adherence to the recommended intake, while some diets, e.g., ketogenic diet, are very difficult to comply with. In addition, cancer cells require fatty acids for energy storage, membrane production, and the generation of signaling molecules (90). Hence, it will be complex to balance fatty acid focused dietary interventions in such a manner that they suppresses tumor vitality instead of promoting tumor growth. Moreover, different cancer types vary in their preferred energy source and metabolic activity. For instance, many cancer types overexpress stearoyl-CoA desaturases (SCD) enzymes (91, 92) which prevents SFA lipotoxicity, and has been suggested to reduce ferroptosis triggered by peroxidation of PUFAs (93). Also, cancer cells frequently upregulate enzymes involved in lipid elongation, which appears to promote cancer progression (94). Additionally, although epidemiological, *in vitro* and preclinical data indicate a potentially beneficial effect of dietary fibers that are fermented into SCFAs, further research would be required to better understand the specificity of the different SCFAs. Furthermore, it is difficult to reach high levels of SCFAs systemically and in peripheral organs via dietary intake. The gut lumen is the major site of production of SCFAs and there is a strong biological gradient for each SCFA from the gut lumen to peripheral organs, which leads to different exposure of cells and tissues to SCFAs (95). Finally, even if sufficiently high systemic levels of SCFAs are reached, it will be essential to prevent comorbidity-related adverse effects such as hyperphagia, hypertriglyceridemia, ectopic lipid deposition in liver and skeletal muscle, and liver and muscle insulin resistance (96).

To ensure clinical application, the direct effects of SCFAs and PUFAs on the immune system and TME and the effects of dietary interventions on the gut epithelial cells and microbiome should be tested. Crucially, the most favorable ratios between different SCFAs, branched SCFAs, saturated, unsaturated and *n*-3 PUFAs/*n*-6 PUFAs, as well as different dosages of the fatty acids should be explored. Human cohort and clinical intervention studies need to be established. If standardized well, these human studies will reveal reliable correlations between the intake of relevant food components and follow-up data from cancer patients receiving immunotherapy, which will help us to better understand the etiology of the responsiveness in patients with different metabolic profiles. To prevent heterogeneity and create robust data, these human clinical interventions studies will also need standardization of protocols (e.g., timing of dietary interventions, timing and dosages of the immunotherapy and fecal and serum samples collection) in combination with detailed multi-analysis. In such well-controlled human clinical trials, baseline and follow-up measurements regarding tumor progression will proof the impact of diet on the outcome of immunotherapy. Moreover, metabolic and biochemical parameters will contribute to the unraveling of the mechanisms underlying the effects of SCFAs and PUFAs on immunotherapy responsiveness in cancer.

Currently, no nutritional biomarkers to predict which patients will respond to immunotherapy are available. Promising epidemiological data do however indicate an association between the patients' nutritional status and immune checkpoint inhibitor therapy efficacy, pointing toward a potential role for fecal and serum SCFA content as well as gut microbiome diversity as biomarker. These data hold promise for the development of biomarker signatures to predict treatment responses, based on metabolic and biochemical data and validated food frequency/lifestyle questionnaires. Most likely, multiple biomarker signatures will be required taking into account subgroup analysis, e.g., patients with obesity will respond differently compared to malnourished patients and therefore need different biomarker signatures. Finally, it will be crucial to validate the developed biomarker signatures in well-controlled human clinical intervention studies as described above.

In conclusion, dietary regimens that focus on SCFAs and PUFAs to improve the outcome of immunotherapeutic approaches hold great promise. Specifically, SCFAs can sensitize cancer cells to immunogenic responses, improve gut barrier function, reduce the cytokine storm and activate cytotoxic T cells. Furthermore, fibers which are fermented into SCFAs can also indirectly influence the outcome of immunotherapy *via* modulation of the gut microbiome. Similar to SCFAs, *n*-3 PUFAs may also reduce the cytokine storm and inhibit chronic low-grade inflammation potentially creating a TME where immune checkpoint inhibitors work more efficiently, whereas other patients may benefit from a diet rich in pro-inflammatory *n*-6 PUFAs actually supporting the immune activating properties of immunotherapy. Despite all the promising data, several challenges remain to be overcome, highlighting the necessity of more studies before dietary interventions focusing on SCFAs and PUFAs can become standard of care in the clinic.

## Author Contributions

JT and RS-S conceived the presented idea. AW and LS wrote the manuscript with support from JB, AH, LD, and RL. All authors contributed to the final version of the manuscript.

## Funding

This work was funded by the NWO domain Applied and Engineered Sciences and Danone Nutricia Research, with additional financial support from Topsector Agri and Food. Grant No. 16485 NutrI2FIT: Strengthening Immune Fitness -a Nutritional solution to boost cancer ImmunoTherapy efficacy to JT. In addition this work was funded by VCK, Grant No. Swu16.0057-VT to RS-S.

## Conflict of Interest

JB and AH are employees of Danone Nutricia Research. The remaining authors declare that the research was conducted in the absence of any commercial or financial relationships that could be construed as a potential conflict of interest.

## Publisher's Note

All claims expressed in this article are solely those of the authors and do not necessarily represent those of their affiliated organizations, or those of the publisher, the editors and the reviewers. Any product that may be evaluated in this article, or claim that may be made by its manufacturer, is not guaranteed or endorsed by the publisher.

## Abbreviations

ARA: arachidonic acid
CRP: C reactive protein
CTLA-4: cytotoxic T-lymphocyte-associated protein 4
DC: dendritic cell
DHA: docosahexaenoic acid
EPA: eicosapentaenoic acid
HDAC: histone deacetylase activity
HFD: high fat diet
HIF-1α: hypoxia-induced factor 1α
HLA: human leukocyte antigen
ICAM-1: intercellular adhesion molecule 1
IFNγ: interferon γ
IL: interleukin
LPS: lipopolysaccharide
LFA: lymphocyte function-associated antigen 1
MDSC: myeloid-derived suppressor cells
MHC-1: major histocompatibility complex 1
MICA/B: MHC class I polypeptide-related sequence A/B
NK: natural killer
NKG2D: natural killer group 2D
NSCLC: non-small cell lung cancer
*n*-3 PUFAs: omega 3 polyunsaturated fatty acids
*n*-6 PUFAs: omega 6 polyunsaturated fatty acids
PD-1: programmed cell death protein 1
PD-L1: programmed death-ligand 1
PNI: prognostic nutritional index
PGE-2: prostaglandin E 2
PUFAs: polyunsaturated fatty acids
SCFAs: short-chain fatty acids
SFAs: saturated fatty acids
TAMs: tumor-associated macrophages
TCR: T cell receptor
TEER: transepithelial electrical resistance
Th1: T helper cell 1
TIGIT: T cell immunoreceptor with Ig and ITIM domains
TME: tumor microenvironment
TNFα: tumor necrosis factor α
TRAIL: tumor necrosis factor-related apoptosis inducing ligand
WHO: world health organization.
