# Supplementary material for: Fatty Acids as a Tool to Boost Cancer Immunotherapy Efficacy
Source: Front Nutr. 2022 Jun 23;9:868436. doi: 10.3389/fnut.2022.868436 (PMC9260274; doi:10.3389/fnut.2022.868436)
Supplement: Supplementary file 1 [file Data_Sheet_1.docx]

Supplementary Material

# Intermezzo box 1

Fatty acids consist of a terminal carboxyl group and a hydrocarbon chain. Fatty acids are often categorized, according to hydrocarbon chain length, into short chain (up to 6 carbons) (SCFAs), medium chain (6 to 12 carbons) (MCFAs), or long chain fatty acids (> 12 carbons) (LCFAs). SCFAs are mostly generated by gut bacteria upon fermentation of indigestible carbohydrates (1). In contrast to SCFAs, MCFAs and LCFAs are mostly taken directly from the diet. In addition to categorizing by hydrocarbon chain length, MCFAs and LCFAs are often classified based on the number of double bonds in the hydrocarbon chain, into saturated (SFAs), monounsaturated (MUFAs), or polyunsaturated fatty acids (PUFAs). Based on the position of the first double bond starting from the methyl terminus, MUFAs and PUFAs are sub-classified in n-3 MUFAs/PUFAs, n-6 MUFAs/PUFAs or n-9 MUFAs/PUFAs respectively.

1. Louis P, Hold GL, Flint HJ. The gut microbiota, bacterial metabolites and colorectal cancer. Nat Rev Microbiol. 2014;12(10):661-72.
